# Risk factors of ICD-11 adjustment disorder in the Lithuanian general population exposed to life stressors

**DOI:** 10.1080/20008198.2019.1708617

**Published:** 2020-01-10

**Authors:** Paulina Zelviene, Evaldas Kazlauskas, Andreas Maercker

**Affiliations:** a Center for Psychotraumatology, Institute of Psychology, Vilnius University, Vilnius, Lithuania; b Division of Psychopathology and Clinical Intervention, Department of Psychology, University of Zurich, Zurich, Switzerland

**Keywords:** Adjustment disorder, stressors, risk factors, ICD-11, psychopathology, Trastorno de adaptación, estresores, Factores De Riesgo, CIE-11, psicopatología, 适应障碍, 压力源, 风险因素, ICD-11, 心理病理学, • The study explored the risk factors of ICD-11 adjustment disorder in the general population.• ICD-11 adjustment disorder was associated with the source of life-stressor; in particular, job-related and health-related stressors were associated with a high risk of adjustment disorder.• ICD-11 adjustment disorder was associated with female gender, greater age, and university education.• The study informs researchers and clinicians about the risk factors of ICD-11 adjustment disorder and provides valuable insights for future research and clinical practice.

## Abstract

**Background**: A new definition of adjustment disorder symptoms has been included in the 11th edition of the International Classification of Diseases (ICD-11). However, little is known about risk factors of ICD-11 adjustment disorder.

**Objective**: The study aimed to analyse risk factors of adjustment disorder in a sample of the Lithuanian general population exposed to life-stressors.

**Method**: In total, the study included 649 adult participants from the general population with various recent significant life-stressor experiences. ICD-11 adjustment disorder symptoms were measured using the Adjustment Disorder New Module-8 (ADNM-8) scale.

**Results**: The prevalence of the ICD-11 adjustment disorder diagnosis in the sample was 16.5%. Job-related stressors and health-related stressors were significantly associated with adjustment disorder. Other risk factors for adjustment disorder in this study were female gender, greater age, and university education.

**Conclusions**: We conclude that stressor type and demographic characteristics are associated with the risk of developing an adjustment disorder.

## Abstract (es)

**Antecedentes**: Se ha incluido una nueva definición de los síntomas del trastorno de adaptación en la 11^a^ edición de la Clasificación Internacional de Enfermedades (CIE-11). Sin embargo, se sabe poco sobre los factores de riesgo del trastorno de ajuste de ICD-11.

**Objetivo**: El estudio tuvo como objetivo analizar los factores de riesgo del trastorno de adaptación en una muestra de la población general lituana expuesta a estresores de la vida.

**Método**: En total, el estudio incluyó a 649 participantes adultos de la población general con varias experiencias recientes de estrés vital significativo. Los síntomas del trastorno de adaptación del CIE-11 se midieron utilizando la escala del Nuevo Módulo 8 del Trastorno del Adaptación (ADNM-8, sigla en inglés).

**Resultados**: La prevalencia del diagnóstico del trastorno de adaptación de la CIE-11 en la muestra fue de 16.5%. Los estresores relacionados con el trabajo y los estresores relacionados con la salud se asociaron significativamente con el trastorno de adaptación. Otros factores de riesgo para el trastorno de adaptación en este estudio fueron el sexo femenino, aumento en la edad y la educación universitaria.

**Conclusiones**: Concluimos que el tipo de estresor y las características demográficas están asociadas con el riesgo de desarrollar un trastorno de adaptación.

## Abstract (zh)

**背景：**适应症症状的新定义已包含在《国际疾病分类》（ICD-11）的第11版中。然而，关于ICD-11调节障碍的危险因素知之甚少。

**目的：**本研究旨在分析暴露于生活压力源的立陶宛普通人群样本中适应障碍的风险因素。

**结果：**样本中ICD-11适应障碍的患病率为16.5％。与工作有关的压力源和与健康有关的压力源与适应障碍显著相关。在这项研究发现适应障碍的其他危险因素是女性, 年龄较大和大学学历。

**结论：**我们总结出压力源类型和人口统计学特征与适应障碍的风险有关。

## 1. Introduction

The definition of adjustment disorder was significantly revised in the 11^th^ edition of International Classification of Diseases (ICD-11) which was released in 2018 by the World Health Organization (WHO) (Zelviene & Kazlauskas, 2018). In ICD-11, adjustment disorder is now recognized as a stress-response syndrome along with other disorders that are specifically associated with stress, such as posttraumatic stress disorder (PTSD), complex PTSD, and prolonged grief disorder. ICD-11 adjustment disorder diagnosis requires an experience of at least one or more identifiable stressors. The symptom profile of adjustment disorder in ICD-11 is defined via two symptom categories of: (1) preoccupation, defined as excessive worry, distressing thoughts and rumination related to the current stressor, and (2) failure to adapt, defined as a significant impairment in important areas of life (social, family or occupational). Symptoms of adjustment disorder should not be of sufficient specificity or severity to justify the diagnosis of another mental and behavioural disorder (World Health Organization [WHO], 2018).

Adjustment disorder is among the most often used mental disorder diagnosis in healthcare. According to WPA-WHO global survey, 50% of surveyed psychiatrists used ICD-10 adjustment disorder diagnosis more than once a week, but at the same time, they identified its low ease of use (Reed, Mendonça Correia, Esparza, Saxena, & Maj, 2011). Despite its frequent use in clinical practice, adjustment disorder has received little attention in research until very recently (Bachem & Casey, 2017; Zelviene & Kazlauskas, 2018). For example, the PubMed, which was conducted in 2017 and looked at publications on adjustment disorder published over a decade, revealed 349 times more research items on depression in comparison to adjustment disorder (Zelviene & Kazlauskas, 2018). The observed neglect of adjustment disorder in research could be explained by previously vaguely defined symptom profile of adjustment disorder (Maercker, Einsle, & Köllner, 2007). It has also been stated that the definition of ICD-10 adjustment disorder was often used as a provisional or residual diagnostic category when the reactions did not fully represent other mental disorders (Maercker et al., 2013). Therefore, new studies, testing the symptom profile of ICD-11 adjustment disorder, its prevalence, and risk factors have emerged following ICD-11 proposals for a new definition (Kazlauskas, Zelviene, Lorenz, Quero, & Maercker, 2017; Maercker et al., 2013).

The most recent studies on ICD-11 adjustment disorder are generally focused on the validation of the symptom structure (Glaesmer, Romppel, Brahler, Hinz, & Maercker, 2015; Kazlauskas, Gegieckaite, Eimontas, Zelviene, & Maercker, 2018; Lorenz, Hyland, Perkonigg, & Maercker, 2017; Lorenz, Perkonigg, & Maercker, 2018a; Zelviene, Kazlauskas, Eimontas, & Maercker, 2017), development of assessment instruments (Bachem, Perkonigg, Stein, & Maercker, 2016; Ben-Ezra, Mahat-Shamir, Lorenz, Lavenda, & Maercker, 2018), development of interventions (Bachem & Maercker, 2016; Eimontas, Rimsaite, Gegieckaite, Zelviene, & Kazlauskas, 2018; Maercker, Bachem, Lorenz, Moser, & Berger, 2015; Moser, Bachem, Berger, & Maercker, 2019; Rachyla et al., 2018), exploring the symptom change (Lorenz, Perkonigg, & Maercker, 2018b; O’Donnell et al., 2016), and possible predictors of ICD-11 adjustment disorder (Lorenz et al., 2018b; Mahat-Shamir et al., 2017; Ring et al., 2018).

Research on ICD-11 adjustment disorder primarily focuses on target populations exposed to specific stressor, such as people who have recently lost their job (Lorenz et al., 2018a), burglary victims (Bachem & Maercker, 2016), survivors of traumatic events, or terror attacks (Mahat-Shamir et al., 2017; Ring et al., 2018). However, several studies also explored ICD-11 adjustment disorder symptoms in the general population, such as Lithuania (Zelviene et al., 2017) or Germany (Glaesmer et al., 2015; Maercker et al., 2012).

The initial studies have reported the prevalence of ICD-11 adjustment disorder ranging from 2% to 29% across several samples. However, the majority of these studies were conducted among high-risk populations exposed to specific stressors. The prevalence of ICD-11 adjustment disorder in representative German population (*N* = 2512) was 2.0% (Glaesmer et al., 2015). The Zurich Adjustment Disorder Study reported 13.8% men and 17.2% women prevalence of ICD-11 adjustment disorder in the sample of individuals who lost their work involuntarily; the prevalence of adjustment disorder increased with higher age and exposure to multiple stressors (Perkonigg, Lorenz, & Maercker, 2018). Another study of burglary victims (*N* = 80) found that 29% of the sample were at high risk for adjustment disorder 21 weeks after the onset of the stressor (Bachem & Maercker, 2016).

Knowledge about the predictors or risk factors of ICD-11 adjustment disorder is lacking. Previous studies found that adjustment disorder may be associated with various life-stressors (Lorenz et al., 2018a; Zelviene et al., 2017), exposure to trauma (O’Donnell et al., 2016; Ring et al., 2018), demographic characteristics and socio-interpersonal factors (Horn & Maercker, 2016; Lorenz et al., 2018a, 2018b). In a community sample of survivors from terror attack in Israel it was found that previous traumatic experiences, stressful events and younger age were significant predictors associated with ICD-11 adjustment disorder symptoms, whereas physical proximity to the site of the terror attack and gender were not (Mahat-Shamir et al., 2017). In the context of involuntary job loss, ICD-11 adjustment disorder was associated with greater age, lower financial household budget, and exposure to multiple stressors and work-related factors (Perkonigg et al., 2018). Interpersonal factors, such as higher loneliness, higher dysfunctional disclosure, and lower self-efficacy were associated with higher adjustment disorder symptom severity and a higher likelihood of meeting the diagnostic criteria for ICD-11 adjustment disorder (Lorenz et al., 2018a).

More information is needed about the distinctive features and risk factors of ICD-11 adjustment disorder. Based on previous empirical studies and the new adjustment disorder ICD-11 symptom profile, we aimed to investigate: (a) the prevalence of adjustment disorder, and (b) the sociodemographic and stressor-related characteristics are associated with ICD-11 adjustment disorder in a non-clinical community sample exposed to various life stressors.

## 2. Method

### 2.1. Participants and procedures

This study was a part of the larger Vilnius Adjustment Disorder Study (VADIS). The methods and procedures of the study have been published previously (Zelviene et al., 2017). The study aimed to collect data from a representative non-clinical sample from across the country using quota-sampling strategies to recruit participants. Data collection took place either at home or at the workplace of the participants using self-report with the assistance of a trained researcher. All procedures of the study were in accordance with the ethical standards of the institutional and national ethical regulations as well as with the 1964 Helsinki declaration and its later amendments or comparable ethical standards. All participants provided written informed consent to participate in the study. The final sample demographic characteristics were similar to the Lithuanian Housing and Population Census data from the year 2011 (Zelviene et al., 2017).

A population-based sample of 831 participants (57.9% women; *M*_age_ = 39.84, *SD*_age_ = 17.83) from various places of residence across Lithuania took part in the study. Only the data of 649 adult participants who indicated having experienced various significant life stressors during the last 2 years and who completed the study were included in the analysis; 182 participants were excluded for not meeting inclusion criteria. The mean age of the study sample was 39.98 years (*SD* = 17.84; range 18–89) of whom 392 (60.4%) were women. 39.4% (*n* = 256) of participants had a university degree, and the majority of 79.0% (*n* = 513) resided in urban areas (Zelviene et al., 2017).

### 2.2. Measures

#### 2.2.1. Adjustment disorder symptoms

The symptom intensity and diagnostic status of ICD-11 adjustment disorder were assessed using the brief Adjustment Disorder New Module-8 (ADNM-8) scale (Kazlauskas et al., 2018), which is a revised version of the ADNM-20 scale (Einsle, Köllner, Dannemann, & Maercker, 2010). The ADNM measure is widely used in ICD-11 adjustment disorder research (Kazlauskas, Zelviene, Lorenz, et al., 2017; Zelviene & Kazlauskas, 2018). The ADNM-8 is a self-report measure comprised of two parts: (1) the stressor list; and (2) the symptom list.

The Lithuanian version of the ADNM-8 stressor list which was used in our study was comprised of 14 life stressors. Participants were also asked to indicate any other stressful events not included among the listed stressors; this item was not included in further analysis. These stressors were further grouped into three categories based on the previous studies which indicated the importance of job-related stressors, interpersonal stressors and health-related stressors for further analysis: (1) four health-related stressors (heart disease, chronic illness, illness of a closed one, other illnesses); (2) three interpersonal stressors (conflicts with family members, divorce/separation, death of the loved one); and (3) seven job-related stressors (conflicts with colleagues, conflicts with superior, unemployment, unexpected job loss, financial problems, adjustment due to retirement, too much or too little work). Participants were asked to indicate stressors that happened during the past one or 2 years and were still a heavy burden at the time of the study or burdened them for the last 6 months. The total score of the ADNM-8 list of life stressors was the sum of all listed stressor items and ranged from 1 to 14. With regards to the three stressor categories, we estimated the presence of a stressor in each stressor-category, health-related, job-related and interpersonal stressors if participant endorsed at least one of the stressors in that category.

The ADNM-8 list of symptoms comprised eight items reflecting the two core ICD-11 adjustment disorder symptoms: (1) preoccupation and (2) failure to adapt, in accordance with the ICD-11 diagnostic criteria for adjustment disorder (WHO, 2018). The four items in the ADNM-8 measured constant preoccupation about the stressor and the thoughts revolving around the stressor. The other four items of the ADNM-8 were used to assess a failure to adapt and measured difficulties to concentrate, sleep disturbances, withdrawal from the close ones, and difficulties in carrying out daily activities or work. Participants were asked to indicate how often the respective symptom items applied to them over the past week on a 4-point Likert scale: 1 = *never*, 2 = *rarely*, 3 = *sometimes*, 4 = *often*. The total score of the symptom part of the ADNM-8 scale was the sum of all the items and ranged from 8 to 32.

Previous studies supported the factor structure of the brief 8-item ADNM-8 measure (Kazlauskas, Zelviene, Lorenz, et al., 2017), and good psychometric characteristics of the ADNM-8 were reported in several studies (Ben-Ezra et al., 2018; Kazlauskas et al., 2018; Lorenz et al., 2018a; Zelviene et al., 2017). In this study, Cronbach’s α was .89 for the total ADNM-8 scale, α = .87 was for the ADNM-8 preoccupation subscale, and α = .79 for the ADNM-8 failure to adapt subscale. In the current study, two-factor confirmatory factor analysis (CFA) yielded a good model fit, CFI/TLI = .971/.958, RMSEA [90% CI] = .063 [.046, .079], SRMR = .032.

We used a cut-off of ≥23 for the total ADNM-8 symptom score to identify a probable ICD-11 adjustment disorder diagnosis based on previous studies of ICD-11 adjustment disorder in Lithuania (Eimontas, Gegieckaite, et al., 2018; Eimontas, Rimsaite, et al., 2018; Skruibis et al., 2016). This ADNM-8 symptom cut-off score was used in two intervention studies for diagnosis and clinical outcome measures of ICD-11 adjustment disorder (Eimontas, Gegieckaite, et al., 2018; Eimontas, Rimsaite, et al., 2018). Participants of this study were allocated to the adjustment disorder group based on this ADNM-8 cut-off of ≥ 23.

#### 2.2.2. Lifetime trauma exposure

Exposure to at least one lifetime traumatic event and accumulative lifetime traumatic experiences were assessed using self-report the Brief Trauma Questionnaire (BTQ) (Schnurr, Spiro, Vielhauer, Findler, & Hamblen, 2002) comprised of 10 potentially traumatic events, such as experiencing or witnessing war zone, a dangerous car accident, technological or natural disaster, serious illness, childhood abuse, physical attack, sexual abuse, serious injury, violent death of a close one, or witnessing life-threatening event. Participants were asked to indicate whether they had experienced a listed event. The total score of the BTQ was the sum of all the items and ranged from 0 to 10. The Lithuanian version of the BTQ has been previously used in several studies in Lithuania (Kazlauskas & Zelviene, 2015, 2017).

#### 2.2.3. Suicide attempt

Participants’ history of previous lifetime suicide attempts was assessed by one item: ‘Have you ever in your life tried to commit suicide?’. Participants were asked to respond with a binary answer ‘*Yes*’ or ‘*No*’ to this question.

### 2.3. Data analysis

The data were analysed using statistical data analysis package IBM SPSS® 25.0. We tested the effects of each of the included variables on adjustment disorder using univariate analysis. Further, we applied multivariate binary logistic analysis with a dependent binary variable of the presence of adjustment disorder with a reference category ‘no diagnosis of adjustment disorder’ to identify predictors of adjustment disorder. All study variables except for the measures of adjustment disorder symptoms and age group were included in the model as independent variables to deal with the potential overfitting of the model (Baybak, 2004). None of the demographic variables were missing. However, 5.5% of the data of the ADNM-8 preoccupation subscale, and 4.9% of the ADNM-8 failure to adapt subscale were missing due to missing responses on one of the items. Missing values were handled by excluding missing cases from the analysis in the applied statistical tests. However, all the available data were used in performing univariate statistical analyses.

## 3. Results

### 3.1. Exposure to stressors and trauma in the sample

All participants in our study experienced significant life stressors over the last two years. The average of reported life stressors in the last two years was 2.44 (*SD* = 1.69), ranging from 1 to 14. One stressor was experienced by 36.5% (*n* = 237), two stressors by 25.7% (*n* = 167), three or more stressors by 37.8% (*n* = 245) of the sample. We found no significant gender effects on the number of stressful events in the sample, women experienced 2.51 (*SD* = 1.62) stressors on average, men – 2.33 (*SD* = 1.78) (*t* (647) = 1.37, *p* = .170).

Almost two thirds, 64.6% (*n* = 419), of the sample indicated exposure to job-related stressors, such as unexpected job loss – 12.8% (*n* = 83), unemployment 9.9% (*n* = 65). Around half, 49.5% (*n* = 321), of the sample reported experience of at least one health-related stressor, such as chronic illness – 19.6% (*n* = 127) or other serious illness – 12.6% (*n* = 82). At least one interpersonal stressor was experienced by 31.1% (*n* = 202) of the sample, such as family conflicts – 24.8% (*n* = 161), divorce/separation – 9.9% (*n* = 64) (see Table 1). Previous suicide attempts were reported by 2.6% (*n* = 16) of the sample, 2 men and 14 women.

**Table 1.** Characteristics of the sample (*N* = 649).

Variables	Total sample (*N* = 649)	Adjustment disorder group (*N* = 107)	Comparison group (*N* = 542)	Significance statistics
Gender				
Male	257 (39.6%)	28 (26.2%)	229 (42.3%)	χ^2^(1) = 9.00**
Female	392 (60.4%)	79 (73.8%)	313 (57.7%)	
Age				
*M* (*SD*)	39.98 (17.84)	45.30 (20.11)	38.93 (17.18)	*t*(138.187) = 3.06**
Age group				
18–29	276 (42.5%)	38 (35.5%)	238 (43.9%)	χ^2^(2) = 9.93**
30–59	264 (40.7%)	40 (37.4%)	224 (41.3%)	
60–89	109 (16.8%)	29 (27.1%)	80 (14.8%)	
Education level				
University degree	256 (39.4%)	49 (45.8%)	207 (38.2%)	χ^2^(1) = 1.86
Other	393 (60.6%)	58 (54.2%)	335 (61.8%)	
Employment status				
Unemployed	300 (46.2%)	56 (52.3%)	244 (45.0%)	χ^2^(1) = 1.64
Employed	349 (53.8%)	51 (47.7%)	298 (55.0%)	
Place of residence				
Urban	513 (79.0%)	83 (77.6%)	430 (79.3%)	χ^2^(1) = 0.08
Rural	136 (21.0%)	24 (22.4%)	112 (20.7%)	
Current stressors, total				
*M* (*SD*)	2.44 (1.69)	3.44 (2.05)	2.24 (1.53)	*t*(130.373) = 5.73***
Interpersonal stressors				
Yes	202 (31.1%)	40 (37.4%)	162 (29.9%)	χ^2^(1) = 2.00
No	447 (68.9%)	67 (62.6%)	380 (70.1%)	
Job-related stressors				
Yes	419 (64.6%)	79 (73.8%)	340 (62.7%)	χ^2^(1) = 4.34*
No	230 (35.4%)	28 (26.2%)	202 (37.3%)	
Health-related stressors				
Yes	321 (49.5%)	76 (71.0%)	245 (45.2%)	χ^2^(1) = 22.82***
No	328 (50.5%)	31 (29.0%)	297 (54.8%)	
Previous suicide attempt				
Yes	16 (2.6%)	7 (7.0%)	9 (1.7%)	F**
No	599 (97.4%)	93 (93.0%)	506 (98.3%)	
Trauma exposure				
Yes	454 (70.0%)	80 (74.8%)	374 (69.0%)	χ^2^(1) = 1.15
No	195 (30.0%)	27 (25.2%)	168 (31.0%)	
Life-time traumatic experiences, *M* (*SD*)	1.65 (1.62)	1.81 (1.72)	1.61 (1.60)	*t*(647) = 1.16
Adjustment disorder symptoms, *M* (*SD*)				
Preoccupation	9.33 (3.41)	14.24 (1.56)	8.29 (2.72)	*t*(263.862) = 30.80***
Failure to adapt	7.07 (2.94)	11.46 (2.21)	6.15 (2.12)	*t*(615) = 23.35***
Total	16.34 (5.80)	25.70 (2.55)	14.32 (4.07)	*t*(239.042) = 37.11***

*F*= Fisher’s Exact Test; *p < .05; **p < .01; ***p < .001.

A substantial proportion of the sample, 70.0% (*n* = 454), reported at least one lifetime traumatic experience. Lifetime traumatic events were significantly associated with life stressors (*r* = .19, *p* < .001). The average number of lifetime traumatic experiences in the study sample was 1.65 (*SD* = 1.62). The most prevalent traumatic events in the sample were: a physical assault 32.2% (*n* = 208), a serious car accident 29.9% (*n* = 194), physical abuse in childhood 23.3% (*n* = 151), other situation in which a respondent was seriously injured or feared being seriously injured or killed 19.1% (*n* = 124).

We found a significant gender effect on trauma exposure. Men reported more traumatic events (*M* = 2.18, *SD* = 1.84) than women (*M* = 1.30, *SD* = 1.36), *t* (435.651) = 6.65, *p* < .001. There was no significant association between trauma exposure and age (*r* = .04, *p* = .323) or between trauma exposure and urban vs. rural place of residence (*t* (185.822) = 1.17, *p* = .245).

### 3.2. ICD-11 adjustment disorder in the sample

Based on the ADNM-8 cut-off score of ≥23 for the total ADNM-8 scale the prevalence of the ICD-11 adjustment disorder diagnosis in the sample was 16.5% (*n* = 107, 28 men and 79 women). The mean score of the total ADNM-8 scale in the adjustment disorder group was 25.70 (*SD* = 2.55). The average levels of the core ICD-11 adjustment disorder symptoms measured with the ADNM-8 subscales in the adjustment disorder group were 14.24 (*SD* = 1.56) for preoccupation symptoms, and 11.46 (*SD* = 2.21) for failure to adapt symptoms (see Table 1). We further explored factors associated with adjustment disorder.

Adjustment disorder group had experienced significantly more life stressors (*M* = 3.44, *SD* = 2.05) in contrast to the comparison group (*M* = 2.24, *SD* = 1.53; *t* (130.373) = 5.73, *p* < .001). The adjustment disorder group reported having experienced significantly more job-related (χ*^2^* (1, *n* = 649) = 4.34, *p* = .035) and health-related (χ^2^ (1, *n* = 649) = 22.82, *p* < .001) stressors (see Table 1). However, we found no significant association between adjustment disorder and the exposure to the interpersonal stressors (χ*^2^* (1, *n* = 649) = 2.00, *p* = .157).

Trauma exposure was not associated with adjustment disorder. There were no significant differences in reported lifetime traumatic experiences between the adjustment disorder group (*M* = 1.81, *SD* = 1.72) and the comparison group (*M* = 1.61, *SD* = 1.60, *t* (647) = 1.16, *p* = .247). Moreover, a chi-square test indicated no significant association between adjustment disorder and exposure to at least one lifetime traumatic experience (χ*^2^* (1, *n* = 649) = 1.15, *p* = .283).

Gender and age were the only significant variables of all demographic characteristics associated with adjustment disorder in our study in univariate analyses. We found a significant gender effect on adjustment disorder (χ*^2^* (1, *n* = 649) = 9.00, *p* < .001). There were significantly more women (73.8%, *n* = 79) than men (26.2%, *n* = 28) in the adjustment disorder group in contrast to the comparison group (57.7% women and 42.3% men).

The mean age in the adjustment disorder group (*M* = 45.30, *SD* = 20.11) was significantly higher than in the comparison group (*M* = 38.93, *SD* = 17.18; *t* (138.187) = −3.06, *p* < .001). More detailed analysis of the association between adjustment disorder and three age groups (18–29 years, 30–59 years, 60+ years) also showed a significant association between adjustment disorder and age. In the adjustment disorder group, a significantly larger proportion of participants were 60+ years old (27.1%) in contrast to the comparison group 14.8% (χ^2^ (1, *n* = 649) = 8.88, *p* < .001).

We found a significant association between adjustment disorder and suicidality. Participants in the adjustment disorder group reported significantly more previous suicide attempts (7.0%, *n* = 7) than in the comparison group (1.7%, *n* = 9) (Fisher’s Exact Test, *p* = .008). University education (χ^2^ (1, *n* = 649) = 1.86, *p* = .173), and reported urban vs. rural place of residence were not associated with adjustment disorder (χ^2^ (1, *n* = 649) = 0.08, *p* = .779).

Multivariate binary logistic regression analysis (R^2^_Nagelkerke_ = .190) revealed that female gender (*OR* = 1.83, *p* = .022), greater age (*OR* = 1.02, *p* = .040), university degree education (*OR* = 1.90, *p* = .015), job-related stressors (*OR* = 1.29, *p* = .012), and health-related stressors (*OR* = 1.86, *p* < .001) were significant predictors of adjustment disorder (see Table 2). Multivariate logistic analysis after correction for multiple testing revealed similar findings to univariate analysis, indicating sociodemographic variables (gender, age, university education) and stressor-related variables (job-related and health-related stressors) as significant risk factors for adjustment disorder. However, previous suicide attempt was not a significant predictor of adjustment disorder in the logistic analysis (see Table 2).

**Table 2.** Multivariate logistic analysis of ICD-11 adjustment disorder predictors (*N* = 649).

Variable	*OR*	*95% CI*	*p*
Female gender	1.83	1.09–3.06	.022
Age	1.02	1.00–1.03	.040
University degree	1.90	1.13–3.18	.015
Unemployment	0.80	0.48–1.32	.376
Rural place of residence	1.21	0.66–2.24	.539
Interpersonal stressors	1.51	0.98–2.32	.060
Job-related stressors	1.29	1.06–1.58	.012
Health-related stressors	1.86	1.43–2.42	< .001
Previous suicide attempt	2.79	0.92–8.45	.070
Trauma exposure	1.65	0.93–2.92	.088

OR = Odds Ratio; 95% CI = Confidence interval.

## 4. Discussion

We analysed the prevalence of ICD-11 adjustment disorder in a large sample of the Lithuanian general population using a novel measure of adjustment disorder symptoms based on the ICD-11 diagnostic criteria. Moreover, it was one of the few first studies to explore the risk factors associated with ICD-11 adjustment disorder.

### 4.1. Prevalence of adjustment disorder

The prevalence of adjustment disorder (16.5%) was high, considering that the sample of the study was non-clinical population exposed to at least one relevant current life stressor. Previous studies reported high prevalence of adjustment disorder (25.6%) in high-risk samples, such as among unemployed individuals (Lorenz et al., 2018a) or in oncological, haematological, and palliative-care settings (Mitchell et al., 2011), and among patients with automatic implantable cardioverter-defibrillator (17%) (Maercker et al., 2007).

High rates of adjustment disorder in the Lithuanian population may be associated with the particular socio-economic and political situation of Lithuania following the societal transition from previous political oppression to an independent country in the European Union (Kazlauskas & Zelviene, 2016). These social changes were associated with increased insecurity of population, high unemployment rates, financial difficulties, and migration of a large proportion of the population. Furthermore, there is a lack of mental services for stress-related disorders in Lithuania (Kazlauskas, Zelviene, & Eimontas, 2017) in comparison to other European countries (Schäfer et al., 2018).

### 4.2. Predictors of adjustment disorder

In our study, several demographic factors predicted the risk for adjustment disorder: female gender, greater age, and university education. Adjustment disorder was also associated with exposure to stressors, particularly those related to employment and health. Our findings are largely in line with other studies that found female gender and elderly age were associated with higher rates of adjustment disorder symptoms (Ayuso-Mateos et al., 2001; Lorenz et al., 2018b). Surprisingly, university education was also found to be a risk factor for adjustment disorder, even though higher education is often considered to be a protective factor for mental disorders. As job-related stressors were associated with adjustment disorder, we could hypothesize that participants of our study with higher education were more exposed to job-related stressors. Our study is in line with other studies that found that job-related stressors, such as higher responsibilities or occupational demanding work positions were associated with ICD-11 adjustment disorder symptoms (Perkonigg et al., 2018). Unemployment in our study was not associated with adjustment disorder in contrast to previous studies that identified high adjustment disorder prevalence associated with job loss (Lorenz et al., 2018b).

Exposure to life-stressors, job- or health-related stressors, was significantly associated with ICD-11 adjustment disorder. We also screened the sample for exposure to trauma and found it did not predict adjustment disorder in our study. It is possible that severe traumatic events, such as car accidents, sexual assaults, interpersonal violence or others, could also trigger job-related or health-related difficulties. This link between exposure to trauma and adjustment disorder has previously been revealed in a longitudinal study in Australia, which found a high prevalence of adjustment disorder (16%) at 12 months after trauma exposure (O’Donnell et al., 2016). However, our study found that only exposure to life stressors was associated with adjustment disorder while exposure to trauma was not, which contributes to the validity of the new definition of ICD-11 adjustment disorder.

Other studies also found an association between suicidal ideation and adjustment disorder (Gradus et al., 2012; Schnyder & Valach, 1997) as well as the relationship between adjustment disorder and self-harming behaviour (Pelkonen, Marttunen, Henriksson, & Lönnqvist, 2005). In our study, the multivariate logistic analysis did not confirm previous suicide attempts as a significant predictor of adjustment disorder, even though the univariate analysis with Fisher’s exact test showed that previous suicide attempts were significantly associated with adjustment disorder. However, the prevalence of suicide attempts in our sample was low amounting to less than 3% and only 16 cases which may explain why we were unable to identify an association between suicide attempts and adjustment disorder due to low statistical power in logistic regression.

### 4.3. Limitations and future directions

Although yielding promising results, the study has several limitations that should be taken into consideration. The cross-sectional design chosen for this study poses one such limitation as adjustment disorder symptoms can change in time. According to the ICD-11 diagnostic definition adjustment disorder resolves within 6 months unless the stressor persists longer (WHO, 2018). Previous studies have suggested that the temporal dimension is an important aspect of adjustment disorder and that it needs further investigation (Lorenz et al., 2018b; O’Donnell et al., 2016). Using the cross-sectional design, we were unable to capture the dynamic nature of adjustment disorder symptoms. Longitudinal studies of adjustment disorder are essential in assessing associations between adjustment disorder and potential risk factors further.

We conducted an analysis of adjustment disorder predictors in the Lithuanian general population with exposure to at least one significant recent life stressor. Our findings showed that specific stressors, such as health-related and job-related stressors were significantly associated with a higher risk of adjustment disorder. However, further studies could focus in greater detail on how the intensity and duration of specific stressors are linked with adjustment disorder. Furthermore, studies that focus on the clinical populations and homogeneous samples characterized by experiences of specific stressors could contribute to a better understanding of the impact of the different life stressors on the symptoms of adjustment disorder. The majority of previous studies of ICD-11 adjustment disorder, including our current study, were conducted among adult populations but there is a growing need for studies of adjustment disorder and its risk factors among children and adolescent populations. Data collection based on self-reporting and questionnaires has its limitations too. Future studies should test our findings using diagnostic interviews. However, there is no available valid diagnostic interview for ICD-11 adjustment disorder yet.

These limitations notwithstanding, we found promising results that are important for further studies in this field. First, our findings indicate that the experience of single identified stressor is rare; we propose to focus on the exploration of the multi-stressor impact on stress responses in future research. Second, longitudinal studies are much-needed and might give a better understanding of the course of symptom development in adjustment disorder in relation to risk/protective factors. Empirical data also show that the majority of the population does not have an adjustment disorder. We propose that studies exploring resilience are also important in the field of adjustment disorder studies.
